# The relationship between knowledge and disaster preparedness of undergraduates responding to forest fires

**DOI:** 10.4102/jamba.v15i1.1408

**Published:** 2023-02-28

**Authors:** Fajar Wulandari, Budijanto Budijanto, Syamsul Bachri, Dwiyono H. Utomo

**Affiliations:** 1 Sekolah Tinggi Keguruan dan Ilmu Pendidikan Singkawang, Singkawang, Indonesia; 2 Department of Geography, Faculty of Social Science, Malang State University, Malang, Indonesia

**Keywords:** relationship, knowledge, student, disaster preparedness, forest fires

## Abstract

Forest and land fires are disasters that regularly occur on the island of Kalimantan, Indonesia. Higher education students on the island of Kalimantan are considered vulnerable to these disasters, hence the need for mandatory disaster knowledge and preparedness for every individual in the area. This research aimed to: (1) determine disaster knowledge and student preparedness in dealing with forest and land fire disasters, (2) determine the relationship between knowledge and student preparedness. This study used a quantitative correlation method with a questionnaire. The data were processed using the Statistical Package for the Social Sciences 21. The research sample used purposive sampling because it followed the needs of the study, which involved 300 students affected by forest fires, representing three universities located in a forest fire–prone area in West Kalimantan province, Indonesia. Each campus has 100 students, a total of 300 students. The results showed as many as 284 students had experienced forest and land fire disasters. In addition, 202 out of 284 students were known to have low disaster knowledge. Four main parameters of disaster preparedness were used to measure student preparedness, namely (1) knowledge and attitudes, (2) emergency response plans, (3) disaster warning system and (4) resource mobilisation. The number of students with high preparedness was 141, while 143 had low preparedness. Thus, student preparedness measures need to be increased to avoid disaster impact.

**Contribution:**

Based on the data analysis, students’ knowledge and preparedness to face forest fires are positively related. It was shown that the higher the learning of students, the higher their readiness and vice versa. It is suggested that there is a need for increasing knowledge and preparedness actions for students in dealing with forest fire disasters through regular disaster lectures, simulations and training so that they can make the right decisions in coping with disasters.

## Introduction

Forest and land fires in Kalimantan occur in carbon-rich peat areas, causing toxic smog (Hayasaka et al. 2014; Siregar 2010). Increased forest fires resulting from human activities contribute to climate change (Abatzoglou & Williams 2016; Aponte, De Groot & Wotton 2016; Carnicer et al. 2022; Kihila 2017), which can be disastrous if not handled properly. Natural disasters and climate change are entering a severe stage to anticipation through education which is a priority in the Sustainable Development Goals (SDGs) (Dzvimbo et al. 2022; King 2011; Kupika, Mbereko & Chinokwetu 2020; Leal Filho et al. 2018; Mawonde & Togo 2019). Various efforts have been made by the government so that land-clearing activities using the traditional slash-and-burn method can be avoided (Solekhan, Kunarto & Febriharini 2019). The slash-and-burn methods normally worsen forest fires. This statement was supported by the Indonesian President Soeharto, on 10 September 1997, through banning the practice of burning forest land in Kalimantan because thick smog had blanketed the territory of Indonesia, Malaysia, Singapore, Brunei, the Philippines and Thailand (Ketterings et al. 1999; Kurniawan 2019). However, land clearing intended for agriculture purposes, such as on oil palm cultivation land (Frimawaty 2020), is allowed and excluded as a criminal offence in Indonesia (Fajrini 2022). Various efforts have been made by the government so that land-clearing forest and land fires in West Kalimantan occur every year and are in a serious stage. Deforestation of the oil palm plantations in Kalimantan is one of the triggers for the emergence of land and forest fires (Arifin, Audina & Putri 2019; Muzdalifah & Yunas 2020), being one of the triggers for the emergence of hotspots that can become a source of fire.

It is known that every year, forest and land fires occur in various provinces in Indonesia, especially in the provinces on the islands of Sumatra and Kalimantan (Suryani 2012; Yusuf et al. 2019). The peatlands spreading from the islands of Sumatra to Kalimantan in Indonesia are quite extensive (Osaki et al. 2016). Peatlands can store large amounts of water, but the surface dries up quickly, making it easy to burn during the dry season. Furthermore, the forest and land fires are caused by the conversion of peat lands for use as oil palm plantations. Approximately 90% of forest land that has been cleared, and agricultural land has been converted into oil palm plantations (Carlson et al. 2013).

The West Kalimantan province experiences forest and land fires every year and experienced an increase from 2015 to 2019, caused by El Niño events, resulting in the largest fires in the last decade (Nurhayati et al. 2021). Peatland fires in West Kalimantan province in 2019 reached 151 919 hectares, thus affecting their potential as air retainers, sources of biodiversity, agricultural production and forest commodities, resulting in thick smog and disrupting residents’ activities (Wahyunto, Supriatna & Agus 2013). Efforts to control fires include detecting hotspots via satellite so that fire sources can be detected early and preventive measures are taken to anticipate forest fires.

West Kalimantan has a high potential for forest fire disasters because of a large number of hotspots and types of peatlands that are easily burned during the dry season (Dicelebica, Akbar & Jati 2022). The cause of the high fluctuation of hotspots from 2015 to 2019, with the highest number in 2015 with 2192 events, was caused by the El Niño phenomenon, which raised the water surface temperature of the sea, causing prolonged droughts and raising land surface temperatures; this is based on research (Yananto & Dewi 2016). The potential for forest and land fires in West Kalimantan is high because of many hotspots and peatlands that are easily burned during the dry season (Dicelebica et al. 2022; Prayoga, Yananto & Kusumo 2017). This statement is supported by research from Bahri (2002), who explained that forest and land fires in Indonesia occur during the dry season in August, September and October, as a month of transition.

The conversion of land prepared for agriculture, oil palm plantations, population growth and increasingly hot climate conditions (Hoscilo et al. 2011; Wulandari & Yanti 2019) are the main causes of regular forest and land fires. Indonesia’s forest and land fires occur every year (Adrianto et al. 2020; Syaufina 2018; Thoha et al. 2019), especially during the dry season. Based on hotspot data obtained from the *Lembaga Penerbangan dan Antariksa Nasional* (LAPAN) [National Institute of Aeronautics and Space] West Kalimantan, in 2019, 26 325 points could become sources of forest fire disasters (LAPAN 2019). In the last 15 years, forest and land fires in West Kalimantan have shown an increasing trend that causes economic, social and environmental losses (Imansyah 2021). Forest and land fires that occurred in 2021 at the end of January have made a record of 52 cases of forest and land fires in West Kalimantan (Kominfo 2021). The latest data in April 2022 detected 408 hotspots spread across the province of West Kalimantan that could cause forest and land fires if not addressed immediately (BMKG 2022).

Disasters pose an urgent threat at the global level, destroying educational centres, health and transportation infrastructure and causing economic losses (Chaudhary & Piracha 2021; CRED 2019; Gonzales & London 2021; Sena & Kifle 2016). Forest and land fires disrupt various activities as well as the health of students in carrying out activities and lectures. Universities in West Kalimantan have also been affected by forest and land fires, which played a role in this research (SAPTOMO, Priyo & Hardjasoemantri 2004; Wulandari & Yanti 2019).

Students, being a vulnerable group, are also at high risk when a disaster occurs (Greer, Wu & Murphy 2018; Nipa et al. 2020) because of their lack of experience in dealing with disasters. On the other hand, universities are located in West Kalimantan, which is prone to forest and land fires. Students who mostly come from various regions have no experience with disasters in the local area (Lovekamp & Tate 2008). Preventive measures are needed to anticipate losses and fatalities from the students.

Disaster knowledge must be applied to anticipate impacts in disaster-prone areas (Dube & Munsaka 2018). Disaster knowledge can raise awareness and understanding of disasters in the local area. Each region has characteristics of potential and vulnerability to disasters. Therefore, disaster knowledge is important in preparing students. Knowledge has been recognised as an important part of disaster management (Kusumastuti et al. 2021). Disaster knowledge consists of several variables, namely knowledge about the location of the incident, knowledge of signs of disaster, knowledge about prevention before it occurs, knowledge when it occurs, and handling after a disaster. Not only knowledge but the element of preparedness is also an important factor for everyone in dealing with disasters.

Disaster preparedness is an action that must be owned by everyone, including students (Wulandari & Yanti 2019) who are included in pre-disaster activities (Bullock, Haddow & Coppola 2013; He & Zhuang 2016). Advanced emergency preparedness and safety can reduce casualties, injuries and general disruption (Ahmad et al. 2018). Preparedness is an effective strategy for preventing the impact of disasters that cause tremendous damage to lives (Rogayan & Dollete 2020; Rokkas, Cornell & Steenkamp 2014; Tan & Xiang 2016). Preparedness in higher education is still very much needed to understand the specific risks of disasters within a particular area (Tkachuck 2016). Preparedness is important for students at universities in disaster-prone areas. Parameters of disaster preparedness are used in research to collect accurate data or information on student preparedness in the face of routine forest fire disasters from the *Lembaga Ilmu Pengetahuan Indonesia* (LIPI) [Indonesian Institute of Sciences]. The preparedness parameters in this study consisted of knowledge and attitudes, emergency response plans, disaster warning systems and resource mobilisation (Sopaheluwakan 2006).

Knowledge is a major factor in the formation of one’s preparedness. Various natural disasters provide lessons on the importance of disaster knowledge so that individuals have the right attitude in making decisions. Knowledge also determines attitudes, making one more alert to disasters that will occur in the environment (Lisnasari 2018). The emergency response plan is an important part of disaster management activities which includes activities related to first aid and safety, and it is hoped that losses can be minimised. Disaster management and response plans should be prepared between individuals and organisations to improve disaster preparedness appropriately (Granberg 2013). The disaster warning system includes warning signs, information distribution and appropriate learning tools. The early warning system includes the design of exercises, simulation exercises, and evaluation of disaster preparedness aimed at protecting self and groups in the event of a disaster. Resource mobilisation, consisting of various resources such as human resources, funds and infrastructure for emergencies, is a crucial factor that either supports or becomes an obstacle to preparedness.

Several related kinds of literature (Aksa et al. 2020b; Bako & Ojolowo 2021; Kusumastuti et al. 2021; Lukman et al. 2021; McFayden et al. 2022; Rukema & Umubyeyi 2019; Tasa & Cakir 2022) present research on disaster preparedness and the role of knowledge in disaster risk reduction (DRR) (Weichselgartner & Pigeon 2015). However, there is limited literature that examines the relationship between the knowledge and preparedness of students in universities located in areas prone to forest and land fires. Other studies, such as Kihila’s (2017), discuss forest fire disaster preparedness in higher education institutions. On the other hand, Kihila (2017) and Wall and Halvorson (2011) discuss forest fire disaster preparedness in universities by implementing active learning strategies. Faiz, Ainuddin and Mukhtar (2015) in Pakistan and Al-Dahash, Kulatunga and Thayaparan (2019) in Iraq discuss research on forest fires in active learning and some weaknesses in disaster preparedness. However, there is limited literature examining the relationship between the knowledge and preparedness of students in dealing with forest fires in disaster-prone areas, so this research is essential.

Based on the limited literature related to student knowledge and preparedness in dealing with forest and land disasters in West Kalimantan province, Indonesia, it is necessary for this study to: (1) determine the knowledge of disaster and student preparedness in dealing with forest fire disasters, and (2) determine student knowledge and preparedness in dealing with forest and land fire disasters in West Kalimantan province, Indonesia.

## Research method

This study used a quantitative method with a correlation approach. The research location was at three universities in the province of West Kalimantan, Indonesia: Tanjungpura University; the *Institut keguruan dan ilmu Pendidikan* (IKIP) [Institute for Teacher Training and Education] of the *Persatuan Guru Republik Indonesia* (PGRI) [Association of Teachers of the Republic of Indonesia], Pontianak; and the *Sekolah Tinggi Keguruan dan Ilmu Pendidikan* (STKIP) [College of Teacher Training and Education], Singkawang. Purposive sampling is used to obtain a correct and appropriate sample and can describe the population used as a research subject based on certain considerations (Sugiyono 2019). The location of this research was chosen because every year, forest and land fires occur which disrupt activities and health, so it is considered important to conduct preparedness research, especially for students. Forest fires in fire-prone areas, such as campuses in research during the dry season, have disrupted activities and health; therefore, it is necessary to conduct disaster preparedness research for students. The three universities are located in peatland areas prone to forest fires. Impacts such as smoke from forest and land fires also interfere with student learning activities. This research was to measure the preparedness and disaster knowledge of students in undergraduate programs at three universities in West Kalimantan province, Indonesia, and their relationships.

The research data obtained were then tabulated according to the category of each variable so that the data appeared more straightforward, concise and easy to understand. For disaster knowledge, the data were categorised according to Table 1, while for student preparedness to face forest fire disasters, they were categorised based on Table 2.

**TABLE 1:** Category level of knowledge.

Index value	Category
67–100	Good
34–66	Sufficient
0–33	Less

*Source*: Triyono. R.B.P., Koswara, A. & Vishnu, A., 2013, Panduan *Penerapan Sekolah Siaga Bencana,* Lembaga Ilmu Pengetahuan Indonesia (LIPI) Pusat Penelitian Geoteknologi, Bandung, viewed 03 April 2022 from, https://anyflip.com/gghu/ytof/basic

**TABLE 2:** Classification of preparedness levels.

Index value	Category
80–100	Very ready
65–79	Ready
55–64	Almost ready
40–54	Less ready
0–39	Unready

*Source*: Sopaheluwakan, J.S., 2006, *Kajian K Esiapsiagaan M Asyarakat Dalam Mengantisipasi Becana Gempa Bumi Dan Tsunami*, LIPI – UNESCO/ISDR [Preprint], Jakarta.

Furthermore, the preparedness of students to face forest and land fire disasters was categorised into five groups of preparedness.

### Data collection

**FIGURE 1:**
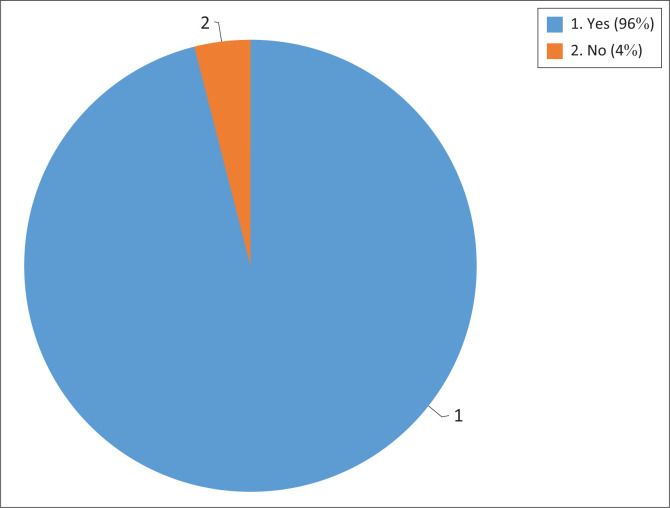
Number of respondents affected by forest and land fires in West Kalimantan province.

A questionnaire was used for data collection from Sopaheluwakan (2006) in Table 3, which contains research instruments designed according to field conditions related to student preparedness in dealing with forest fire disasters. The questionnaires were distributed to 300 students from three universities in West Kalimantan province, Indonesia. This study adopted a purposive sampling technique in selecting respondents. Students, who were the overall research subjects, also experienced the impact of the forest and land fires, which can be seen in Figure 1.

**TABLE 3:** Knowledge instruments and student preparedness in facing forest and land fire disasters.

Variable	Indicator	Question
Preparedness	Knowledge and attitude	10
	Emergency response plan	4
	Disaster warning system	8
	Resource mobilisation	3
Knowledge disaster	Disaster prediction	2
	Disaster prevention	2
	Disaster preparedness and prevention training are needed	2
	Disaster education is required for students.	2
	Natural disasters, social disasters, non-natural disasters	2

**Total**	**-**	**35**

The questionnaire used in this study consisted of two variables: disaster preparedness and disaster knowledge. The first variable is based on preparedness parameters from (Sopaheluwakan, 2006), with four preparedness parameters, namely knowledge and attitudes, with ten questions. The second parameter is an emergency response plan with four questions. The third parameter, namely the early warning system, has eight questions, and the last is resource mobilisation, with three questions shown in Table 4.

**TABLE 4:** Main research questions for students.

Variable	Research instrument
**Preparedness indicators**	
Knowledge and attitude	A natural disaster is an event that suddenly or without warning causes losses in the form of material, property, and loss of life. Forest fires and smoke haze during dry seasons resulting from land clearing by burning. The fires in Indonesia are caused by intentional and negligent human activities. The impact of forest fires and smoke shortens visibility, cause respiratory and other diseases, and are sources of dangerous pollutants. Forest fire is a condition in which forest and land fires occur, which result in forest and land damage, cause economic and environmental losses and disrupt activities and public health. Forest and land fires usually occur in peatland areas. Haze from forest and land fires is influenced by two main factors: climatic conditions and human activities in the forest, garden, and land management. Actions are taken in the forest fire disaster evacuation plan. Burning forests and land creates smoke. Burnings are carried out in particular and controlled areas, while locations affected by forest and land fires are scattered and uncontrolled and cross-regional boundaries.
Emergency response plan	Actionable emergency response plans. It is necessary to conduct emergency response exercises and simulations regarding forest and land fire disasters. The dangers of forest and land fires need to be introduced to students. Rehabilitation and reconstruction plans are needed after forest fire disasters.
Early warning system	Establish a standard process and the roles and responsibilities of all organisations that issue alerts and are mandated by law. Establish agreements and interagency protocols to ensure the consistency of hazard warnings and communication channels where different agencies handle different hazards. Rescue routes and evacuation directions must be placed in the campus and community. Testing and training the warning system throughout the system is carried out at least once a year. Warning centres such as Kamling posts need to be implemented in the community and guarded by the community or staff, either at night or around the clock. Store essential and emergency numbers such as police station phone numbers, hospital phone numbers, *Badan Penanggulangan Bencana Daerah* (BPBD) [Regional Disaster Management Agency] phone numbers, Search and Rescue phone numbers and family phone numbers or other emergency phone numbers. Develop a hazard map for forest and land fires in the Singkawang City area. Need lectures, syllabi, modules and textbooks that contain material for disaster and student preparedness in dealing with forest and land fire disasters.
Resource mobilisation	Students must be involved in preparedness measures to prevent the risks of disasters. Students, lecturers, and related parties participated in socialising preparedness for forest and land fire disasters. Simulation and training for prevention and rescue action to cope with forest and land fire disasters were conducted twice a year.
**Disaster knowledge indicator**	
Disaster prediction	Disasters can be known when they occur. Disasters occur in disaster-prone areas.
Disaster prevention	Protect the environment, do not throw away cigarette butts, and do not clear land by burning forests Use water appropriately, especially in the dry season.
Disaster preparedness and training	Understand disaster management measures through the disaster-related learning material. Carry out training, simulations, and disaster response programs regularly, such as proper firefighting measures.
Mandatory disaster education	Provide and provide disaster-related learning materials for all students who are in the disaster-risk area. Organise extracurricular and compulsory disaster education in every education unit in disaster-prone areas.
Types of disasters (natural, social, and non-natural)	Natural disasters, social disasters, and non-natural disasters are types of disasters. Floods are natural disasters, social conflicts are social disasters, while pandemics such as COVID-19 are non-natural disasters.

Meanwhile, the disaster knowledge parameter for students has ten questions, of which the indicators are disaster prediction, disaster prevention, disaster preparedness and training, mandatory disaster education, and various types of disasters (natural, social, and non-natural); each has two questions as seen in Table 3.

As a mean to the number of questions in this research, the questionnaire is 35 questions to explore preparedness and knowledge data from students in disaster-prone areas.

### Data analysis

This research was conducted in April 2022, using a quantitative questionnaire designed for adaptation to forest fire–prone areas distributed to students at three universities in West Kalimantan province for feedback. The survey is based on questions discussing disaster knowledge and disaster preparedness and the responses used to find the relationship between disaster knowledge and disaster preparedness.

This study adopted a purposive sampling technique, namely a sampling technique with specific considerations to select universities and respondents. Civil students in this study were 100 respondents who represented each university. West Kalimantan province was the research area because it experiences and is affected by forest fires yearly. In addition, all three also experienced the severe impact of forest fires. The data of the two variables were then analysed by Pearson bivariate correlation using the Statistical Package for the Social Sciences (SPSS) 21 (IBM Corporation, Armonk, New York, United States). Then they were processed based on statistical analysis so that the results of disaster knowledge and student preparedness were obtained.

## Research results and discussion

The need for disaster education in DRR in universities is a way to reduce the impact of disasters on students (ERMA 2021). Students need knowledge for resilience to face disasters (Nakanishi & Black 2018). This study provides information on disaster knowledge and students’ preparedness to confront forest and land fire disasters in West Kalimantan province, Indonesia. Respondents in this study were students from various regions studying at three universities in West Kalimantan province. The following illustrates the number of students who have experienced and been affected by forest and land fire disasters.

Based on Figure 1, students who have experienced and been affected by forest and land fire disasters were 96% or 284 students of the total number of respondents, while those who have not experienced it were 12 people or as many as 4%. The number of students who have experienced the effects of forest and land fires in West Kalimantan province directly is higher because of natural conditions and weather that have increased temperatures so that forest fires are routine and cover a relatively large area.

In line with the above incident, several studies have stated that forest and land fires occur during the dry season because of increased temperature and heat, thereby increasing the risk of forest and land fires (Liu, Ballantyne & Cooper 2018). In addition, land clearing and human activities exacerbate fires and their impacts (Jawad, Nurdjali & Widiastuti 2015). Figure 1 shows that almost all students have experienced forest fires, which are seasonal disasters in their area. This incident should warn of the importance of preventive measures, disaster preparedness and transfer of knowledge through disaster knowledge so that forest and land fires do not become disasters that can cause casualties and material losses.

### Disaster knowledge and preparedness

Knowledge of disaster risk in the region is an important and basic need for students. Knowledge is the main aspect that must be considered in improving disaster preparedness (Sopaheluwakan 2006; Takahashi et al. 2015). Disaster knowledge is categorised into three categories, namely good, sufficient and less. Table 5 presents the results of disaster knowledge of students from three universities located in areas prone to forest and land fires in West Kalimantan province, Indonesia.

**TABLE 5:** Categories of disaster knowledge.

Index value	Category	Student	Percentage
67–100	Good	164	58
34–66	Sufficient	110	39
0–33	Less	10	4

**Total**	**-**	**284**	**100**

The results of the analysis are in Table 5, which illustrates that 284 student respondents have experienced forest fire disasters. There are three groupings of disaster knowledge, most of which are in the ‘good’ category, as much as 57%; then 39% have sufficient disaster knowledge, while those with less knowledge are less than 5%. This result is caused by the lack of materials and lectures related to disasters, so only a few students have general knowledge about these catastrophes.

The finding that no compulsory courses are available in higher education also hinders students from understanding disasters. Socialisation and training in dealing with disasters are not provided, even though the area is located in an area prone to forest fires, so naturally, many students’ knowledge about disasters still needs to be improved. Efforts to overcome these limitations may be made by building a knowledge platform for students (Bhandari & Takahashi 2022), training (Juanita, Suratmi & Maghfiroh 2018) and simulation to improve disaster preparedness (Gunawan et al. 2019). In line with this, according to research from Nipa et al. (2020), educational institutions such as universities do not have emergency programs to prepare students for disasters. Applying disaster risk education to the university curriculum (Aksa et al. 2020a) increases student preparedness to face disasters. Consequently, students understand disaster preparedness in the location.

### Student preparedness in facing forest and land fire disasters

This section describes the results of student preparedness in dealing with forest and land fire disasters based on preparedness parameters, including knowledge and attitudes, emergency response plans, disaster warning systems and resource mobilisation (Table 6).

**TABLE 6a:** Student preparedness for forest and land fires.

Preparedness parameter	Very ready	Ready	Almost ready	Not ready	Least ready	Total	Parameter index	Category
Knowledge and attitude	105	62	50	13	54	284	69	Ready
Emergency response plan	60	79	0	45	100	-	63	Almost ready
Disaster warning system	72	76	0	34	102	-	68	Ready
Resource mobilisation	0	164	0	120	0	-	56	Almost ready
Preparedness combined index score	-	-	-	-	-	-	63	Almost ready

**TABLE 6b:** Maximum weight data per parameter

Number	Preparedness parameter index				Total
	KA	EP	WS	RMC	
1	10	4	8	3	25

KA, knowledge and attitudes; EP, emergency response plan; WS, disaster warning system; RMC, resource mobilisation.

The student preparedness data obtained in Table 6 above was calculated using the following formula (Sopaheluwakan 2006), the combined index of ‘preparedness’ with data for each parameter:


$$\begin{array}{l} Preparedness\;Composite\;index=\left(\frac{real\;weight\;KA}{\max\;weight}x\;Index\;KA\right)+\\ \left(\frac{real\;weight\;EP}{\max\;weight}x\;Index\;EP\right)+\left(\frac{real\;weight\;WS}{\max\;weight}x\;Index\;WS\right)+\\ \left(\frac{real\;weight\;RMC}{\max\;weight}x\;Index\;RMC\right)Preparedness\;Composite\;index\\ =\left(\frac{10}{25}\times 69\right)+\left(\frac{4}{25}\times 4\right)+\left(\frac{8}{25}\times 25\right)+\left(\frac{3}{25}\times 25\right)\\ =28+10+18+7\\ =63. \end{array}$$ [Eqn 1]

The combined preparedness index in Table 6 shows a value of 63. Furthermore, as seen in Table 2, the classification of preparedness categories is included in the ‘almost ready’ category.

The results in Table 6 show the combined preparedness index value for all parameters of 63, which is included in the ‘almost ready’ category, even though it is at the threshold value. Furthermore, each parameter has a different preparedness index value according to the number of scores for each respondent (student).

The knowledge and attitude parameters have an index value of 69 with the ‘ready’ category. Respondents on this parameter also indicate that the majority have index values in the ‘very ready’ and ‘ready’ categories of more than 160 respondents. However, there were still more than 50 ‘less ready’ respondents. There were still respondents who could only answer 2 items out of 10 questions on this parameter. The average respondent on this parameter was only able to answer seven items. Overall, the correct answers from the respondents relate to the impact of the fire disaster and several aspects of fire prevention attitudes and actions.

The second parameter is the emergency response plan, which has an index value of 63 and is categorised as ‘almost ready’. This second parameter has an index value that is still lacking, because 145 respondents could be said to not understand the emergency response plan if a disaster occurs. As explained in the emergency management strategy (Public Safety Canada 2019), there is a need for a strategy or plan for each individual to cope with disaster emergencies in areas where disasters frequently occur. On average, respondents could only answer one to two questions from all questions. Respondents have not correctly answered the question of roles and plans in dealing with disasters.

The third parameter of the disaster warning system is included in the ‘ready’ category, with an index value of 68. However, this parameter is the same as the second parameter, because more than 135 respondents had low readiness. Only 72 respondents were ‘very ready’, and 0 were ‘almost ready’. On average, respondents correctly answered four of the eight questions, and respondents did not know the warning centre and evacuation routes because of the lack of warning information related to disasters.

The last parameter of resource mobilisation was the lowest of the other parameters, with an index value of 56, and was included in the ‘almost ready’ category. In this parameter, there were no respondents in the ‘very ready’ category, although there were 164 respondents in the ‘ready’ category, and as many as 120 respondents were ‘not ready’. On average, respondents could only answer one question correctly out of the three. Meanwhile, most questions were answered correctly, namely the importance of training and simulations that discuss disasters.

Based on four indicators of disaster preparedness for students in West Kalimantan, who are in areas prone to forest and land fires, they are in the ‘almost ready’ category. This matter was indicated by the value of the preparedness index reaching 63. The preparedness index value is a combination of the four indicators. Knowledge and attitude indicators are essential in increasing student preparedness because they can adapt and increase their knowledge to be ready to face disaster (Kalanlar 2018; Tuladhar et al. 2014; Wiwik Astuti, Werdhiana & Wahyono 2021). However, this is different from research (Nakano & Yamori 2021) which explains that increasing knowledge does not always indicate a change in students’ readiness to face disasters because there may still be an indifferent attitude, so they are not ready. Thus, if students have high disaster knowledge and preparedness, they can make decisions and act in rescue in the event of a disaster.

### Relationship knowledge and preparedness

The location of universities in areas prone to forest and land fires requires that every individual be prepared and alert, because disasters can occur without notification. Knowledge is required for preparedness and disaster risk reduction (Albris, Lauta & Raju 2020; Kamil et al. 2020). The importance of knowing the relationship between knowledge and student preparedness at three universities in West Kalimantan province, Indonesia, was tested using the Spearman correlation (Table 7).

**TABLE 7:** Analysis of the relationship between knowledge and preparedness.

Variables	Knowledge	Preparedness
**Knowledge**		
Pearson correlation	1	0.202**
Sig. (2-tailed)	-	0.001
*N*	284	284
**Preparedness**		
Pearson correlation	0.202**	1
Sig. (2-tailed)	0.001	-
*N*	284	284

** , Correlation is significant at the 0.01 level (2-tailed).

Based on the analysis results presented in Table 7, the significance value (2-tailed) was 0.001 < 0.05, so there is a positive and significant relationship between knowledge and preparedness for students in dealing with forest and land fires in West Kalimantan province, Indonesia. In addition, the correlation coefficient value of 0.202 shows the relationship between knowledge and preparedness. Data on the relationship between knowledge and preparedness explains that the higher students’ knowledge of disaster, the higher the preparedness of students to face forest and land fires. The knowledge results align with the preparedness that exists in students. If knowledge is high, student preparedness is also high. However, if knowledge is low, preparedness is also low; this is also supported by research conducted by Lukman et al. (2021); Mathew, Schulenberg and Lair (2018); Patel, Kermanshachi and Nipa (2020); Sujarwo, Noorhamdani and Fathoni (2018); and Wiwik Astuti et al. (2021). Other studies explain that there must be readiness from institutions such as universities, because disasters are beyond human control. Student preparedness needs to be strengthened by providing materials or lectures related to science (Berhanu et al. 2016), but it can also be strengthened by providing training, preparedness simulations or meetings related to disasters so that students are always ready in line with research (Abulebda et al. 2020; Pearson 2011).

## Conclusion

Forest fires that occur every year in West Kalimantan province have turned into disasters, especially for universities located in vulnerable areas, which have caused material and nonmaterial losses. Furthermore, there is an interest in developing strategies to prevent land clearing by burning, and plans for students and universities to be prepared in the face of forest fires that may reoccur. This study examines the relationship between disaster knowledge and student preparedness in dealing with forest fire disasters. Knowledge and preparedness are interrelated with each other. Overall, there is a positive relationship between the variables of knowledge and preparedness. The higher the student’s knowledge, the higher the readiness and vice versa: the lower the ability, the lower the disaster preparedness. Students very much need materials and learning about disaster and preparedness. It also reveals that the four preparedness parameters must be fully prepared.

In these cases, significant improvements are needed to strengthen emergency preparedness. Individual awareness efforts to protect the environment must be increased by avoiding land clearing by burning land. In addition, lectures need to be supported by discussing disaster materials and training on preparedness actions for students in forest fire–prone areas. In summary, this study demonstrates the urgent need to address the lack of knowledge and readiness in universities in forest fire-prone areas in West Kalimantan province. They must provide materials or lectures related to disaster knowledge and strengthening training, preparedness simulations or disaster-related meetings to prepare students to face forest fire disasters and the surrounding environment.
